# Supplementary material for: Patient–clinician dynamics in remote consultations: a qualitative study of cardiology and rheumatology outpatient clinics in the UK
Source: BMJ Open. 2023 May 30;13(5):e070923. doi: 10.1136/bmjopen-2022-070923 (PMC10254951; doi:10.1136/bmjopen-2022-070923)
Supplement: Supplementary data [file bmjopen-2022-070923supp001.pdf]

Remote consultations- Rheumatology

Interview schedule - patient

Thank you for agreeing to take part in this project. As you'll have read in the information document, we are interested in your experience and views of remote rheumatology consultations. So, in this call, I'll ask you some questions about these consultations – there are no right or wrong answers, we're just interested in your opinions. I'll record what is said in this call so that it can be typed up later, but any information that could identify you, such as names of people or places, will be removed. If at any time during the call you would like to stop, just let me know, and you do not have to answer any questions that you do not feel comfortable with.

Before we begin, do you have any questions?

[Start recorder]

To start with, can you tell me how many remote (telephone) rheumatology consultations you have had (roughly)?

And when did the remote consultations start? How long had you been having rheumatology appointments before the COVID pandemic?

Can you describe for me what typically happened when you attended the clinic for face-to-face consultations?

How does the session begin? Do you do any preparation before the session?

What information do you try to give the clinician?

How does the session end?

Do you feel the length of the sessions was appropriate (initial session = 30 mins, FU = 15 mins)

Can you describe for me what typically happens in your remote consultations?

How does the session begin? Do you do any preparation before the session?

What information do you try to give the clinician?

How does the session end?

What are the most important differences for you?

Did you receive a clinic letter informing you of your remote consultation?

Do you tend to read everything in your clinic letters or focus on, say, the appointment time?

What, if anything, have you found difficult in conducting remote consultations?

Expressing what is wrong?

Understanding instructions/advice?

Building rapport with clinician?

Technology difficulties – poor lines, hard to take videos of problem area etc.

Has anything helped you to overcome these difficulties?

For example, has asking certain questions helped you to gain a better understanding of what the clinician needs to know/your condition?

Support from partner/friend during the call?

Has anything about the remote consultation been better for you than when meeting in person?

Convenience? More relaxed at home? Not having to take time off work? No travel/parking costs? No waiting at the hospital?

Have you had any face-to-face consultations recently/since having remote consultations?

If yes, how did you find these?

Would you have any concerns if the clinic were to keep running consultations remotely (beyond COVID), at least some of the time?

For your level of care or safety?

Are some types of consultations better conducted remotely than others?

If the clinic was to start using video call, rather than telephone, for remote consultations, how would you feel about this?

If there are times when your remote consultation works particularly well, what do you think made the difference?

And what sorts of things make you feel a consultation hasn't gone very well?

Are there any situations where you would prefer a remote consultation?

For example, drug education, disease education, medication review.

Is there any support or training, for clinicians, other staff or for you, that you think would improve remote consultations?

For example, access to more advanced technology, guidance for clinicians on effective consultations?

That's all my questions, is there anything else you would like to add?

Thank them for their time and stop recorder.

Interview schedule - clinician

Thank you for agreeing to take part in this project. As you'll have read in the information document, we are interested in your experience and views of remote rheumatology consultations. So, in this call, I'll ask you some questions about these consultations – there are no right or wrong answers, we're just interested in your opinions. I'll record what is said in this call so that it can be typed up later, but any information that could identify you, such as names of people or places, will be removed. If at any time during the call you would like to stop, just let me know, and you do not have to answer any questions that you do not feel comfortable with.

Before we begin, do you have any questions?

[Start recorder]

To start with, can you tell me how you used to conduct typical face-to-face outpatient consultations?

What preparation would you do before the consultation? (e.g. look at PROMs, screening responses)

How would you start the consultation?

What information would you try to gather from the patient? (likely to differ depending on reason for consultation – what are the differences?)

Are there any visual clues you look for in a face-to-face consultation, e.g., in the way a patient walks into the room or sits down?

How would you try to end the consultation?

Can you tell me how you would conduct a typical remote consultation?

What preparation would you do before the consultation?

How would you start the consultation?

What information would you try to gather from the patient?

How would you try to end the consultation?

What do you feel are the main differences between remote and typical face-to-face consultations from your perspective?

Are there differences in length between remote and typical face-to-face consultations?

Have you noticed any differences in how patients respond in remote versus face-to-face consultations?

What, if anything, have you found difficult in conducting remote consultations?

Inability to conduct clinical examination or conduct further tests?

Rapport building? Expressing empathy? Breaking bad news?

Understanding patients' descriptions/building a clear picture of the problem without being able to see properly? Assessing whether patients have understood the conversation?

Certain types of conditions or patient groups unsuitable for remote consultations?

Technology difficulties – poor lines, fuzzy pictures etc

Has anything helped you to overcome these difficulties or to improve your remote consultations?

Asking certain questions helped you to gain a better understanding of the patients' conditions?

Have you asked patients to perform their own clinical examinations, e.g. using the DAS (disease activity score) app?

Technology support improved connections?

Discussing or debriefing with colleagues about cases or approaches using this format?

How have you found the use of PROMs to assess patients' suitability for telephone consultations?

Do they provide sufficient information?

Any concerns?

Are there any advantages to remote consultations compared with consultations run in person?

Time saving? Patients more relaxed/comfortable in their own homes?

Would you have any concerns about the rheumatology department continuing with, and potentially increasing the amount of, remote consultations in the long-term?

For patient care, confidentiality or safety?

For clinical workload? Emotional exhaustion?

Would you be happy for more consultations to be conducted remotely in future (beyond the COVID pandemic)? Why/why not? Any provisos?

What do you think are the markers of a successful or good quality remote consultation?

Do these differ from the markers of a successful face-to-face consultation? How?

And what would characterise a poor quality remote consultation?

What outcomes do you think it is important to measure during/after remote consultations? Why?

e.g. number of painful joints/the sound of the person's voice in terms of engagement/energy levels/concern about symptom control or questions raised by the patient about ongoing care

Is there any support or training, for clinicians or patients, that you think would improve the quality of remote consultations?

Are the protocols you have for face-to-face appointments (e.g., breaking bad news etc) fit for purpose with remote consultations?

Help for patients in setting up video-call equipment, access to more advanced technology, guidance for clinicians on effective consultations?

That's all my questions, is there anything else you would like to add?

Thank them for their time and stop recorder.
