# Supplementary material for: Patient–clinician dynamics in remote consultations: a qualitative study of cardiology and rheumatology outpatient clinics in the UK
Source: BMJ Open. 2023 May 30;13(5):e070923. doi: 10.1136/bmjopen-2022-070923 (PMC10254951; doi:10.1136/bmjopen-2022-070923)
Supplement: Supplementary data [file bmjopen-2022-070923supp002.pdf]

Standards for Reporting Qualitative Research - checklist

SRQR item	Manuscript page number (line number, if applicable)
Item 1. Title	1 (1-2)
Item 2. Abstract	2-3 (1-23, 1-2)
Item 3. Problem Formulation	5
Item 4. Purpose or research question	5 (21-23)
Item 5. Qualitative approach and research paradigm	6-8
Item 6. Researcher characteristics and reflexivity	8-9
Item 7. Context: Setting/site and salient contextual factors; rationale	5 (15-23) and 6 -7 (18-2)
Item 8. Sampling strategy	7
Item 9. Ethical issues pertaining to human subjects	6
Item 10. Data collection methods	7-8
Item 11. Data collection instruments and technologies	7-8, plus Supplementary file 1
Item 12. Units of study	8
Item 13. Data processing	8
Item 14. Data analysis	8
Item 15. Techniques to enhance trustworthiness	8
Item 16. Synthesis and interpretation	9 - 18
Item 17. Links to empirical data	Throughout Discussion, pages 18-23
Item 18. Integration with prior work, implications, transferability, and contribution(s) to the field	Throughout Discussion, pages 18-23
Item 19. Limitations	22
Item 20. Conflicts of interest	None to declare, 23 (20)
Item 21. Funding	23 (22)
