# Supplementary material for: Patient–clinician dynamics in remote consultations: a qualitative study of cardiology and rheumatology outpatient clinics in the UK
Source: BMJ Open. 2023 May 30;13(5):e070923. doi: 10.1136/bmjopen-2022-070923 (PMC10254951; doi:10.1136/bmjopen-2022-070923)
Supplement: Author's manuscript [file bmjopen-2022-070923.draft_revisions.pdf]

BMJ Open is committed to open peer review. As part of this commitment we make the peer review history of every article we publish publicly available.

When an article is published we post the peer reviewers' comments and the authors' responses online. We also post the versions of the paper that were used during peer review. These are the versions that the peer review comments apply to.

The versions of the paper that follow are the versions that were submitted during the peer review process. They are not the versions of record or the final published versions. They should not be cited or distributed as the published version of this manuscript.

BMJ Open is an open access journal and the full, final, typeset and author-corrected version of record of the manuscript is available on our site with no access controls, subscription charges or pay-per-view fees (http://bmjopen.bmj.com).

If you have any questions on BMJ Open's open peer review process please email info.bmjopen@bmj.com

BMJ Open

Patient-Clinician Dynamics in Remote Consultations: A Qualitative Study of Cardiology and Rheumatology Outpatient Clinics

Journal:	*BMJ Open*
Manuscript ID	bmjopen-2022-070923
Article Type:	Original research
Date Submitted by the Author:	15-Dec-2022
Complete List of Authors:	Grey, Elisabeth; University of Bath, Department for Health; University of Bristol Medical School<br>Brown, Frankie; University of Bath, Department for Health<br>Smith, Paula; University of Bath - Claverton Down Campus, Department of Psychology<br>Springett, Daniella; University of Bath, Department for Health<br>Augustine, Dan; Royal United Hospitals Bath NHS Foundation Trust; Royal United Hospitals Bath NHS Foundation Trust<br>Sengupta, Raj; The Royal National Hospital for Rheumatic Diseases, Bath<br>Peacock, Oliver; University of Bath, Department for Health<br>Gillison, Fiona; University of Bath, Department for Health
Keywords:	Quality in health care < HEALTH SERVICES ADMINISTRATION & MANAGEMENT, QUALITATIVE RESEARCH, RHEUMATOLOGY, CARDIOLOGY

SCHOLARONE™
Manuscripts

I, the Submitting Author has the right to grant and does grant on behalf of all authors of the Work (as defined in the below author licence), an exclusive licence and/or a non-exclusive licence for contributions from authors who are: i) UK Crown employees; ii) where BMJ has agreed a CC-BY licence shall apply, and/or iii) in accordance with the terms applicable for US Federal Government officers or employees acting as part of their official duties; on a worldwide, perpetual, irrevocable, royalty-free basis to BMJ Publishing Group Ltd ("BMJ") its licensees and where the relevant Journal is co-owned by BMJ to the co-owners of the Journal, to publish the Work in this journal and any other BMJ products and to exploit all rights, as set out in our licence.

The Submitting Author accepts and understands that any supply made under these terms is made by BMJ to the Submitting Author unless you are acting as an employee on behalf of your employer or a postgraduate student of an affiliated institution which is paying any applicable article publishing charge ("APC") for Open Access articles. Where the Submitting Author wishes to make the Work available on an Open Access basis (and intends to pay the relevant APC), the terms of reuse of such Open Access shall be governed by a Creative Commons licence – details of these licences and which Creative Commons licence will apply to this Work are set out in our licence referred to above.

Other than as permitted in any relevant BMJ Author's Self Archiving Policies, I confirm this Work has not been accepted for publication elsewhere, is not being considered for publication elsewhere and does not duplicate material already published. I confirm all authors consent to publication of this Work and authorise the granting of this licence.

For peer review only

Patient-Clinician Dynamics in Remote Consultations: A Qualitative Study of Cardiology and Rheumatology Outpatient Clinics

Elisabeth Grey[a.b*], Frankie Brown[b], Paula Smith[c], Daniella Springett[b], Dan Augustine[b, d], Raj Sengupta[e, f], Oliver Peacock[b] and Fiona Gillison[b]

[a] Bristol Medical School (Population Health Sciences), University of Bristol, Bristol, UK

[b] Department for Health, University of Bath, Bath, UK

[c] Department of Psychology, University of Bath, Bath, UK

[d] Royal United Hospitals Bath NHS Foundation Trust, Bath, UK

[e] Royal National Hospital for Rheumatic Diseases, Bath, UK

[f] Department of Pharmacy and Pharmacology, University of Bath, Bath, UK

*Corresponding author email: e.b.grey@bath.ac.uk

Word count: 4763

For peer review only

For peer review only - http://bmjopen.bmj.com/site/about/guidelines.xhtml

1

For peer review only

For peer review only - http://bmjopen.bmj.com/site/about/guidelines.xhtml

Abstract

Objective: Explore the experiences of patients and clinicians in rheumatology and cardiology outpatient clinics during the first year of the COVID-19 pandemic, focusing on the impact of remote consultations on interpersonal dynamics.

Design: Qualitative phenomenological design, using semi-structured interviews conducted between February and June 2021, and analysed using thematic analysis.

Setting: The rheumatology and cardiology departments of a general hospital in England, UK.

Participants: All clinicians and a convenience sample of 100 patients in each department who had taken part in a remote consultation in the past month were invited to take part. Twenty-five interviews were conducted (13 with patients, 12 with clinicians).

Results: Three themes were explored: adapting to the dynamics of remote consultations, impact on the patient, and impact on clinicians. Both clinicians and patients found remote consultations to be more business-like and focused, with the absence of pauses restricting time for reflection. For patients with stable, well-managed conditions, remote consultations were felt to be appropriate and could be more convenient than in-person consultations. However, the loss of visual cues meant some patients felt they could not give a holistic view of their condition and limited clinicians' ability to gather and convey information. Clinicians adjusted their approach by asking more questions, checking understanding more frequently, and expressing empathy verbally, but felt patients still shared fewer concerns remotely than in person; a perception with which patients concurred.

Conclusions: These findings highlight the importance of ensuring, for each patient, that remote care is appropriate. Future research should focus on developing ways to support both clinicians and patients to gather and provide all information necessary during remote consultations, to enhance communication and trust.

For peer review only - http://bmjopen.bmj.com/site/about/guidelines.xhtml

Article summary

Strengths and limitations of this study

- This study provides novel insight into the ways in which clinician-patient communication is experienced in remote consultations, providing greater depth to the existing evidence on practical advantages and disadvantages of this care format.
- This is one of few qualitative studies to consider clinicians' experiences of remote consultations alongside patients', identifying that the challenges of this format are recognised by both.
- Participants were recruited from two departments of one general hospital, limiting the generalisability of the findings.
- This study focused specifically on experiences during the first year of the COVID-19 pandemic – acceptance and expectations for remote consultations is likely to evolve over time as people become more familiar with this format and technology advances.

Keywords: remote consultations; patient experience; clinician experience; interpersonal communication

For peer review only - http://bmjopen.bmj.com/site/about/guidelines.xhtml

Introduction

The COVID-19 pandemic precipitated the rapid implementation of remote consultations in both primary and secondary care [1] and remote delivery of health services is set to continue. In the UK, for example, NHS planning guidance for 2022 sets out the ambition that "at least 25% [of outpatient appointments] should be delivered remotely by telephone or video consultation (equivalent to c.40% of outpatient appointments that don't involve a procedure)" [2]. Research conducted prior to the COVID-19 pandemic can inform us on user experience when planned remote services are implemented, but the rapid rise in the use of technology during the pandemic means that the background level of skill and acceptability has changed. Recent research has pointed to a need for the post-pandemic remote delivery model to evolve in both primary [3] and secondary care [4] to reflect the variation in and changing levels of patients' and clinicians' confidence and skills, as well as changes in technology.

Research has highlighted some of the advantages and disadvantages of remote consultations for outpatient care during the COVID-19 pandemic. Routinely collected feedback reported advantages, such as reduced stress, enhanced accessibility, cost, and time savings [4], and disadvantages including technical difficulties [3-5] and the inability to conduct physical examination [6-8]. Remote consultations are also reported to challenge communication due to the lack of visual cues, such as body language and facial expressions, in telephone consultations (e.g., to gauge patient understanding) [6 9 10] and reduced cues in the case of videocalls (e.g., not able to see the patients' movement in and out of a consultation room) [10].

Relatively limited qualitative research is available exploring some of these difficulties in more depth. Analysis of three studies incorporating interviews with 35 patients with musculoskeletal conditions found acceptability of the consultation format varied independently of characteristics of the condition alone [10]. Clinicians have reported barriers such as a lack of confidence in patients' ability to

For peer review only - http://bmjopen.bmj.com/site/about/guidelines.xhtml

communicate remotely and patients endorsed clinicians' views that remote consultations are not as satisfactory when building new relationships [10]. Gilbert and colleagues (2022) reported how interpersonal relations influence the acceptability of video consultations in orthopaedics, suggesting the norms and expectations for remote consultations of both staff and patients play an important role in determining satisfaction with a consultation.

As remote consultations are set to remain a part of health service delivery, it is important to understand how they impact patient-clinician dynamics (that is, the interaction between patient and clinician) and identify ways to ensure they fully meet the needs of both. In this phenomenological qualitative interview study, we explored patient and clinician experiences of remote consultations conducted during the first year of the COVID-19 pandemic from two outpatient settings in a UK hospital that deliver care for people with chronic conditions: rheumatology and cardiology. Our focus was on the impact that remote consultations have on interpersonal dynamics and the individuals involved when compared with in-person consultations.

Methods

Design

Data were gathered using in-depth semi-structured interviews conducted by telephone or online call between February and June 2021. The interview topic guide was developed by the research team, including collaborating clinicians, and informed by a rapid review of published literature on the acceptability, advantages and disadvantages of delivering remote consultations in secondary care (conducted on published research up to February 2021). The interview schedules comprised open-ended questions and prompts to elicit detailed responses, with questions focusing on participants' experiences of face-to-face and remote consultations, markers of good quality remote consultations, and barriers and facilitators to successful remote consultations. Separate interview schedules were

For peer review only

For peer review only - http://bmjopen.bmj.com/site/about/guidelines.xhtml

developed for patients and staff, and for rheumatology and cardiology, to ensure the question wording was appropriate for participants' experience. An example interview schedule for rheumatology patients is provided in Supplementary file 1. This study received ethical approval from the Research Ethics Approval Committee for Health at the University of Bath (Reference Number: EP 20/21 008).

Participants and Recruitment

Clinicians: Emails were sent to all clinicians in patient-facing job roles working in participating departments, inviting them to take part in the study and providing a link to an online information sheet and consent form. The invitation came from clinical colleagues but it was made clear that the interviews and analysis would be conducted independently by the research team and participation would be anonymous.

Patients: Patients who had completed at least one remote consultation in participating departments in the past month were eligible to take part. A sample of 100 eligible patients in each of the two departments was identified and invitation letters and information sheets were sent to them in the post; invitations directed patients to either complete an online consent form or contact the research team directly if they were interested in taking part.

Procedure

Participants first completed an online form to provide, first, consent and, second, demographic information including age group, gender, ethnic group, length of time receiving treatment from the department (patients), and professional role and length of time in profession (clinicians). For some patients, consent and demographic data were collected at the start of the interview calls. Semi-structured interviews were conducted remotely, either by telephone or video call, by two researchers (EG and DS).

For peer review only

Patient and public involvement

Due to the lockdown restrictions in place at the time of study planning and the short time limit in which to complete the study for the funders, we were unable to involve patients and the public in the design and conduct of the study. Findings were, however, reported to patient involvement groups at the hospital and their reflections on these helped to inform a further study.

Analysis

Interviews were audio recorded and transcribed intelligent verbatim. Personal, identifying information was removed from transcripts prior to analysis. Transcripts were analysed thematically [11 12] using NVivo version 12 software to help organise, code and explore the data. EG and PS, both experienced qualitative researchers with a background in health psychology, first read a subset of the transcripts to familiarize themselves with the data; EG then coded the subset and compiled an initial coding framework, in discussion with PS. EG and DS (a doctoral student, supervised in analysis by EG) then coded the remainder of the transcripts adding new codes where necessary, continuing to discuss and refine the framework with PS. The final codes were organised into themes by EG and discussed with the research team. An inductive approach to generating codes and themes was taken in the initial analysis, but the selection and development of final themes was driven by the aim of focusing on the psychosocial aspects of consultations.

Results

In total, 25 interviews were conducted: 12 with clinicians (six cardiology and six rheumatology) and 13 with patients (three cardiology and ten rheumatology). Due to the low numbers involved, overall demographic data for the sample are reported here to protect clinicians' and patients' identities. The clinician participants from each department included both nurses (N = 5) and doctors (N = 7), seven were male and the majority (75%) were white, with 6 to 46 years of experience in the

For peer review only - http://bmjopen.bmj.com/site/about/guidelines.xhtml

profession. All patients were white, the majority (67%) were female and ranged in age group from

Theme	Key points
Adapting to the dynamics of remote consultations	Remote consultations tend to be more focused, “business-like” - this can make it harder to build a rapport and mean patients do not raise all their concerns<br>The lack of visual cues in telephone consultations can mean it takes longer to gather/convey information<br>Relying on oral communication to convey empathy, reassurance, encouragement etc. is not always satisfactory<br>The lack of visual cues in telephone consultations makes it harder to assess patients’ understanding
Impact on the patient	The focused style and lack of visual information leaves some patients feeling unable to convey a holistic view of themselves

35-44 years to over 75 years. Patients had been receiving treatment from their respective departments for between two months and over 20 years. Most remote consultations conducted in this hospital were by telephone, although a few cardiology clinicians and patients had also conducted video-call consultations.

Clinicians and patients alike felt the dynamic of remote consultations would be suitable for regular, routine follow-up appointments where the patient’s condition was stable; they were considered unsuitable for initial appointments and breaking bad news. Remote consultations were considered to offer advantages in terms of convenience and improving access for patients who live far away from the hospital, who struggle to get time off work for an appointment or who struggle to afford the travel costs. Three themes were developed that went beyond this feedback on the practicalities of remote consultations to explore the interpersonal effects of moving to a remote model of care; the key points for each theme are summarised in Table 1. The first, largest theme, which was consistent across both patient and clinician participants, was *Adapting to the dynamics of remote consultations*. Theme two focused on the *Impact on the patient* of remote consultations and the final theme was on the *Impact on clinicians.* The same topics and perspectives were raised by interviewees from both departments, so are presented together within each theme.

For peer review only
For peer review only - http://bmjopen.bmj.com/site/about/guidelines.xhtml

	The physical presence of a clinician can be reassuring; the lack of it can leave patients feeling less well cared for and uncertain of their clinician's level of concern
Impact on clinicians	Relying on only oral communication to gather and convey information can be exhausting<br>In the majority of cases clinicians are confident of their ability to accurately assess and treat patients remotely, but there is a heightened fear of missing important signs when not seeing patients face-to-face<br>The diminished social contact of remote consultations may be less satisfying to clinicians

Table 1. Summary of key points within each theme

Theme 1: Adapting to the dynamics of remote consultations

Clinicians and patients both reported that remote consultations tended to be more focused, with less 'chat'.

> *Patients don't tend to chat as much either interestingly. People I know who are chatty, and I have to keep on pulling them on track when I'm face to face with them, are much more focused on just what they need to tell me. The actual interview tends to take less time, because they don't side track themselves so much when they're on the phone. (RC03, rheumatology nurse, female)*

This was attributed by some to the different conventions associated with telephone calls compared to those of meeting people in person, with telephone calls being more typically used for short or functional exchanges of information.

> *They seem to have been briefer and quicker because we both know the difficulties. I'm kind of used to dealing with technology so I'm used to the way you use it, you talk, they talk … it's a very precise form of communication. (RP12, rheumatology patient, male)*

There was some indication that in face-to-face appointments this 'chat' provides a function of establishing rapport and trust and may also contribute to setting a more relaxed tone in which

For peer review only

For peer review only - http://bmjopen.bmj.com/site/about/guidelines.xhtml

patients feel comfortable raising things that they worry are less important (explored further in Theme 2).

There's something about the phone that means that I'm also more business-like and think 'oh no I won't waste time asking that', and maybe brush over things a bit more. Whereas if I was sitting in a consultation and actually looking at the consultant, I might open up a little bit more. (CP06, cardiology patient, female)

While this more focused dynamic could work well for patients whose conditions were stable and who had an established relationship with the clinicians, it made it harder to develop a rapport when the clinician and patient had not previously met.

I would never ever want to meet a person first time over the phone. I think it's always good to look somebody in the eye properly over a table rather than virtually. And then it's a lot easier to have problem phone calls afterwards because you've met them, you know them. You get a sense for somebody, I think. (CP07, cardiology patient, male)

Having to make calls from unknown numbers was also felt to present a barrier to establishing trust.

I think on a telephone consult it's quite difficult to get across who you are… because our numbers are withheld from the hospital you're taking it for granted that they trust you. Whereas if you've got somebody in a room you build that rapport immediately, I think, by introducing yourself and having eye contact and that kind of thing. (RC01, rheumatology doctor, male)

While remote consultations were perceived to be more focused, clinicians often reported taking longer to question patients as they needed to find new ways of gathering the information needed for treatment decisions that would previously have been evident through visual cues.

For peer review only

For peer review only - http://bmjopen.bmj.com/site/about/guidelines.xhtml

Obviously, you can tell whether somebody looks well or unwell when they walk in... Whereas obviously I don't know that on the telephone, so I have to ask them. You can tell that a bit from the way they talk and what they're talking about, and the energy in their voice, or the lack of energy in their voice. So yeah, there is a little bit of that that you don't see, so you have to ask more questions. (CC04, cardiology doctor, male)

Clinicians discussed how they tried to create the space for the discussion of additional questions or concerns in remote consultations, just as they would in face-to-face consultations, to try and encourage greater communication when consulting remotely. They also explained how they tried to use their own body language in face-to-face consultations to express empathy, reassurance or encouragement; while they found means of expressing these orally in remote consultations, this was not always felt to be effective, particularly for 'stoical' patients.

Because you can use your own body language to actually encourage them to talk, which is really hard to do over the phone; there's only so many ways you can rephrase things until they get fed up with you. (RC03, rheumatology nurse, female)

Sometimes they're not prepared to talk to you over the phone about it. Particularly if you don't know them very well, some people are still reluctant to tell you everything... Some of them are very stoical, our patients. (RC03, rheumatology nurse, female)

The missing visual cues in remote consultations also made it harder for clinicians to assess patients' understanding of the information and advice discussed, again leading them to ask more questions.

I find that I tend to repeat myself quite a lot to check if I'm getting through to them, because I can't assess nodding or smiles or visual clues to know that I'm making sense. So, I constantly say "I hope that's clear", "I hope that makes sense to you". So, it's a bit

For peer review only - http://bmjopen.bmj.com/site/about/guidelines.xhtml

longwinded I find, some of my consultations, because I keep checking that they've understood what I'm trying to say. (CC01, cardiology doctor, male)

Clinicians also relayed how visual cues from the patient in face-to-face consultations were relied upon for determining subtler aspects of their (i.e., the clinician's) conduct, such as how to pitch the level of information or their tone of voice.

The way they look at you, whether they look in your eyes or whether they don't look at you at all. That can reflect them being scared a lot of the time, or maybe not understanding. (CC04, cardiology doctor, male)

You can tell how somebody feels by looking at their face and sometimes you can tell if there's something else going on that they want to talk about by looking at them and seeing how they are. You can also tell if somebody's very happy with how they are being treated by their body language. That's been one of the challenges that I've found with telephone consults, is knowing how somebody is just by being around them potentially. (RC01, rheumatology doctor, male)

Despite clinicians tending to ask more questions in remote consultations, the focused dynamic meant there was also a tendency not to leave pauses. Breaks in conversation during consultations are important for allowing patients time to think or to process bad news together and the lack of pauses in remote consultations was felt by both patients and clinicians.

I think it's hard because in a face-to-face consultation you can have pauses, where you're thinking about things… It feels less pressured. Telephone conversations are often 'I talk and then you talk', but there's not much time for a pause or reflection of what has been said. And then it's over, and you can't. It's like often if you finish a consultation, you go to the door and you sort of say "Oh, what about this, can you just tell me quickly about this

For peer review only

For peer review only - http://bmjopen.bmj.com/site/about/guidelines.xhtml

before I go?". Whereas with a phone call once it's over, it's over and that's it." (CP09, cardiology patient, female)

If you've just got time just to sit and accept the silence together that's sometimes part of it, and on a telephone you're always going to try and fill that awkward silence. Whereas if they're there with you, you don't have to, you can just let that go and then just wait. (CC04, cardiology doctor, male)

Theme 2: Impact on the patient

When patients were not physically seen by clinicians, some felt that they couldn't convey a full, holistic view of themselves. Patients reported finding it hard to find the right words to discuss their condition, or they could not or did not want to talk about more indirect consequences of their condition or treatment such as weight gain or fatigue. In a face-to-face consultation, they felt the clinician would be able to see these without them needing to raise the topic first themselves

When you're not meeting with them, they're just on the phone, it doesn't feel like they're totally across it. Because they haven't seen you, they haven't examined you. They don't know what you look like… she could probably tell by looking whether I'm in a bad phase or a good phase or whether the bad phases are worse than they used to be. (RP08, rheumatology patient, female)

I think their intuition does play a part in medicine, with doctors… there's nothing quite like sitting face-to-face seeing what colour the skin is, what the general feeling is, what the energy is like. (RP16, rheumatology patient, female)

As one patient pointed out, this may be partially remedied by having video rather than telephone consultations.

For peer review only - http://bmjopen.bmj.com/site/about/guidelines.xhtml

Sometimes if it's [a clinician] I don't know they'll say, "do you manage to look after yourself?" … hopefully through a video they can see a bit of my home and how busy life is It gives the impression you feel they are on my wavelength a bit faster. That I don't have a carer coming in!" (RP09, rheumatology patient, female)

The physical presence of a clinician in face-to-face consultations was a clear sign that someone was looking after them and also enabled patients to benefit from the visual cues in a clinician's body language (e.g., when expressing empathy or concern).

To see them and be reassured by their demeanour - because you can tell when you look at people whether they're concerned about something or not. They might reassure you verbally, but you can pick up if they think 'oh no this is something we need to investigate further'. (CP09, cardiology patient, female)

The diminished reassurance in remote consultations seemed particularly to be felt when patients were suffering worse symptoms or experiencing more uncertainty.

It would be peace of mind as well, because if the consultant says "I don't think there's any need [for treatment]", at least I feel that they have seen my ailment… if it was me, personally, in pain, I really feel I need to see- I would want to see them face-to-face. (RP15, rheumatology patient, female)

Theme 3: The impact of remote consultations on clinicians

Clinicians reported that the additional effort needed to gather information, develop rapport and convey interest and understanding solely through speech, without seeing patients, could be exhausting.

For peer review only

Telephone consults can be quite draining though because you end up talking quite a lot. So, I think if you're doing telephone consults non-stop day in, day out, then from a clinician point of view it would be quite draining. (RC01, rheumatology doctor, male)

When they have got tender swollen joints it's just really difficult - it's a long drawn out conversation to identify where the problems are if people have got multiple troublesome joints. If it's just a couple that's not really a problem, but if they're having a lot of problems, I just find that a challenging conversation. (RC02, rheumatology nurse, female)

Without being able to see patients or conduct physical examinations, clinicians also reported feeling a fear that they might be missing important signs of disease or misdiagnosing patients. Although in the vast majority of cases clinicians were confident in their ability to accurately assess and treat patients remotely, there could nonetheless be a background-level doubt, particularly for 'stoical' patients.

There's always going to be that slight niggle at the back of your mind about the patient who thinks they are doing very well but actually they've got some gradual progressive disease that they are not aware of. (RC06, rheumatology doctor, male)

You will always have a clue, there will be blood tests and the letter ... But you do worry, that there might be someone that, because you haven't seen them, that you miss a diagnosis or you don't take on board the severity of a diagnosis. (CC02, cardiology doctor, male)

One clinician also highlighted the potential for safeguarding issues to arise when they couldn't see patients, again causing uncertainty for staff.

I have had a situation… calling somebody's mobile phone and their partner answering and saying that they were too unwell to come to the phone. And then that brings up like

For peer review only - http://bmjopen.bmj.com/site/about/guidelines.xhtml

a safeguarding issue - is that patient at risk? If they're so unwell that they can't come to the phone… Are they actually unwell? (RC01, rheumatology doctor, male)

Not being able to see patients also meant that clinicians missed an important source of positive feedback, such as patients smiling or acting more at ease following a successful course of treatment. This, in addition to the diminished rapport, made remote consultations less sociable and satisfying for clinicians.

It's definitely, in some ways, less satisfying because you can't see the patient smile. And sometimes when patients are walking out the clinic room, you have a bit of banter... There isn't that additional kind of interaction which you get from a face-to-face consult. (CC01, cardiology doctor, male)

I do think we are visual. I think we do relate to people better when we can see them… I prefer that myself, rather than over the phone. (CC05, cardiology nurse, female)

Discussion

This paper reports on the interpersonal impacts of remote consultations for clinicians and patients in the year following the onset of the COVID-19 pandemic. Both clinicians and patients perceived a shift in the tone of consultations to be more "business-like" and focused when delivered remotely rather than in person, attributing this to the conventions of telephone calls. Both patients and clinicians flagged the absence of pauses in conversations, removing time for reflection on information delivered, particularly when this was bad or unwelcome news. Clinicians reported having adjusted their approach by asking more questions, checking understanding more frequently and finding ways to express their own understanding and empathy verbally where they would previously have relied on body language. Some found this more tiring than face-to-face consultations. Even with such adaptations, there was acceptance among clinicians that patients

For peer review only - http://bmjopen.bmj.com/site/about/guidelines.xhtml

seemed less comfortable discussing certain topics and disclosing as much information as they would have done in person; this may be a particular concern among 'stoic' patients. Supporting clinicians' perceptions, patients reported being less likely to raise 'small' issues in remote consultations that they would have done in face-to-face consultations and felt that they had been less able to communicate a holistic version of themselves.

The majority of research on remote consultations in secondary care has focused only on the experiences of patients [10 13]; the present study adds to the small body of research that incorporates both patient and health care professional perspectives, which provides an opportunity to explore reciprocal or interacting effects. A mixed methods study conducted in a UK outpatient department following the rapid implementation of a remote video consultation platform indicated that, while all patients reported that the consultations met their needs, patients were less satisfied than clinicians that they had communicated everything they wanted to (86% vs 95%) [14]. A quarter of clinicians showed some recognition of this, reporting a belief that patient experience of a remote video consultation was worse than a face-to-face clinic [14 15]. Similarly, an interview study with patients and clinicians at an orthopaedic centre in the UK found some clinicians perceived video call assessments to be less accurate and more likely to result in worsening of a patients' condition; this corresponded to some patients' experiencing slower rehabilitation and perceiving remote appointments to be less effective than face-to-face [15]. Among our sample of cardiology and rheumatology clinicians, we also found concerns that their patients were not as willing or able to communicate as fully in remote consultations as when face-to-face and an awareness that they do not get a comprehensive picture without seeing patients in person, leaving them uncertain as to whether there is more they should be doing for their patients. Thus, research suggests that patients and clinicians, when communicating remotely, can feel a lack of certainty that either they or the other party has read the situation correctly. This can contribute to perceiving remote consultations to be poorer quality than traditional face-to-face consultations.

For peer review only - http://bmjopen.bmj.com/site/about/guidelines.xhtml

Implications

The present study has confirmed that while remote consultations are acceptable and can provide a basis for care, they do not provide a like-for-like exchange [16]. Past work has flagged the need to match the format of consultations to different types of appointment (e.g., remote consultations are less appropriate for initial appointments and breaking bad news, but may work well for the follow-up of stable cases [17]), but there may also be benefit in matching the format to types of patient (e.g., stoic, health anxious, higher risk) and, potentially, to preferences among clinicians. Practical means of identifying how to enable flexible delivery when responding to these variations when implementing at scale may be essential for the successful incorporation of remote consultations into future mainstream care.

Given that some degree of remote care is certain to continue [16], further work is needed to find effective ways of enabling patients to give, and clinicians to gather, all necessary information during remote consultations. This could help to set the expectations for and, in turn, satisfaction with remote consultations of both patients and clinicians [15]. This may involve, for example, additional training for clinicians in communication techniques for remote consultations, different appointment structures to allow space for reflection, and exploring other approaches that could help to improve both patient and professional confidence and satisfaction. Similarly, educational resources could be developed for patients to provide advice on how they can best prepare and encourage them to mention both major and minor issues and to use their full allocated time slot rather than stick to usual "business-like" norms for phone contact.

As both patients and clinicians become more familiar with remote consultations, long-term research will be useful in identifying changes (or not) in the impact of technological restrictions, comfort with communicating online, clinician job satisfaction and other unanticipated consequences of greater remote provision. While patients were generally happy to accept remote care during the peaks of

For peer review only - http://bmjopen.bmj.com/site/about/guidelines.xhtml

the pandemic, seeing it as preferable to no care or to increasing their risk of infection by visiting the hospital, this may have changed now that everyday life is increasingly less restricted.

Limitations

The participants in the present study are from a limited pool of respondents from a regional hospital in South West England. The hospital draws patients from a wide range of socio-economic backgrounds as well as both rural and urban contexts, but with little ethnic diversity. It should also be noted that our sample did not include young adults (<35 years), who are likely to have different acceptance levels and expectations for remote health communication [18]. Therefore, a wider range of considerations and influences may have been discussed if conducted in hospitals based in other communities. While we invited 100 patients from both departments, this was much more successful with rheumatology patients, so these are more strongly represented. Most remote consultations within the hospital were conducted via telephone. This is not unusual across the NHS, due to both the availability of equipment and private space for clinicians, as well as concerns about patient access to technology and digital skills. Video consultations are likely to have a different dynamic but were were experienced by only two participants in this study. Finally, these interviews took place during a lockdown period and before vaccines were available free of charge for all adults. As such, acceptance of the need to attend remotely was likely higher among both patients and clinicians than when concerns about the risk of infection are lower.

Conclusions

The use of remote consultations in secondary outpatient care is largely accepted by patients and clinicians, and set to remain in future healthcare delivery. While technical difficulties in the delivery of remote consultations are rapidly being overcome, more attention is needed on identifying ways to ensure remote consultations are employed appropriately and that, when they are, patients and

For peer review only - http://bmjopen.bmj.com/site/about/guidelines.xhtml

clinicians are best enabled to communicate effectively. Remote consultations may never be able to fully replicate the interpersonal environment of face-to-face consultations, but research can help to identify ways to ensure that important elements of consulting are not lost and may also highlight traits of remote communication that are particularly suited to certain patients or clinicians.

Author contributions

EG, FB, FG and PS were responsible for the study conception and design. EG and DS conducted the interviews and coded the transcripts; EG was responsible for developing the coding framework and thematic analysis with input from PS. EG composed a first draft of the manuscript with contributions from FB and FG. All authors commented on the draft manuscript. All authors read and approved the final manuscript.

Acknowledgements

We would like to thank all patients and clinicians who participated in this study. We are also grateful to the health professionals who provided feedback on the findings in departmental workshops.

Ethics approval and consent to participate

The protocol was approved by the Research Ethics Approval Committee for Health at the University of Bath (Reference Number: EP 20/21 008). All methods were carried out in accordance with the relevant guidelines and regulations. Informed consent was obtained and documented electronically from all participants prior to each interview.

Competing interests

None to declare.

Funding

For peer review only - http://bmjopen.bmj.com/site/about/guidelines.xhtml

For peer review only

This work was supported by the UKRI Strategic Priorities Fund: Evidence Based Policy Making theme.

Availability of data and materials

The datasets generated and analysed in this study are not publicly available to reduce the risk of breaking the confidentiality of the study participants, but are available from the corresponding author on reasonable request.

For peer review only

For peer review only - http://bmjopen.bmj.com/site/about/guidelines.xhtml

References

1. Gilbert AW, Billany JCT, Adam R, et al. Rapid implementation of virtual clinics due to COVID-19: report and early evaluation of a quality improvement initiative. *BMJ Open Qual* 2020;9(2) doi: 10.1136/bmjoq-2020-000985
2. NHS England. 2021/22 priorities and operational planning guidance: October 2021 to March 2022. https://www.england.nhs.uk/wp-content/uploads/2021/09/C1400-2122-priorites-and-operational-planning-guidance-oct21-march21.pdf, 2021.
3. Murphy M, Scott LJ, Salisbury C, et al. Implementation of remote consulting in UK primary care following the COVID-19 pandemic: a mixed-methods longitudinal study. *Br J Gen Pract* 2021;71(704):e166-e77. doi: 10.3399/BJGP.2020.0948 [published Online First: 20210225]
4. Bradwell H, Baines R, Edwards KJ, et al. Exploring Patient and Staff Experiences With Video Consultations During COVID-19 in an English Outpatient Care Setting: Secondary Data Analysis of Routinely Collected Feedback Data. *JMIR Form Res* 2022;6(3):e30486. doi: 10.2196/30486 [published Online First: 21.3.2022]
5. Gilbert AW, Mentzakis E, May CR, et al. Patient preferences for use of virtual consultations in an orthopaedic rehabilitation setting: Results from a discrete choice experiment. *J Health Serv Res Policy* 2021:13558196211035427. doi: 10.1177/13558196211035427 [published Online First: 2021/08/03]
6. Isautier JM, Copp T, Ayre J, et al. People's Experiences and Satisfaction With Telehealth During the COVID-19 Pandemic in Australia: Cross-Sectional Survey Study. *J Med Internet Res* 2020;22(12):e24531. doi: 10.2196/24531 [published Online First: 20201210]
7. Jones MT, Arif R, Rai A. Patient Experiences With Telemedicine in a National Health Service Rheumatology Outpatient Department During Coronavirus Disease-19. *J Patient Exp* 2021;8:23743735211034973.
8. Sloan M, Lever E, Harwood R, et al. Telemedicine in rheumatology: A mixed methods study exploring acceptability, preferences and experiences among patients and clinicians. *Rheumatology (Oxford)* 2021 doi: 10.1093/rheumatology/keab796 [published Online First: 20211026]
9. Liberati E, Richards N, Parker J, et al. Remote care for mental health: qualitative study with service users, carers and staff during the COVID-19 pandemic. *BMJ Open* 2021;11(4):e049210. doi: 10.1136/bmjopen-2021-049210 [published Online First: 20210422]
10. Paskins Z, Bullock L, Manning F, et al. Acceptability of, and preferences for, remote consulting during COVID-19 among older patients with two common long-term musculoskeletal conditions: findings from three qualitative studies and recommendations for practice. *BMC Musculoskeletal Disorders* 2022;23(1):312. doi: 10.1186/s12891-022-05273-1
11. Braun V, Clarke V. Using thematic analysis in psychology. *Qualitative research in psychology* 2006;3(2):77-101.
12. Braun V, Clarke V, Hayfield N, et al. Thematic analysis. 2019:843-60.
13. Tyler JMB, Pratt AC, Wooster J, et al. The impact of increased outpatient telehealth during COVID-19: Retrospective analysis of patient survey and routine activity data from a major healthcare system in England. *The International Journal of Health Planning and Management* 2021;36(4):1338-45. doi: 10.1002/hpm.3185
14. Dhahri AA, Iqbal MR, Pardoe H. Agile Application of Video Telemedicine During the COVID-19 Pandemic. *Cureus* 2020;12(11):e11320. doi: 10.7759/cureus.11320 [published Online First: 20201104]
15. Gilbert AW, Jones J, Stokes M, et al. Patient, clinician and manager experience of the accelerated implementation of virtual consultations following COVID-19: A qualitative study of

For peer review only - http://bmjopen.bmj.com/site/about/guidelines.xhtml

preferences in a tertiary orthopaedic rehabilitation setting. *Health Expectations* 2022;25(2):775-90. doi: https://doi.org/10.1111/hex.13425

16. Mann C, Turner A, Salisbury C. The impact of remote consultations on personalised care: Evidence briefing. Available from: https://arc-w.nihr.ac.uk/Wordpress/wp-content/uploads/2021/08/Remote-consultation-briefing-website-final.pdf: NIHR ARC West, 2021.

17. Chan A, Suarez A, Kitchen J, et al. Teleclinics in rheumatology introduced during the first lockdown phase of the COVID-19 pandemic of 2020. *Future Healthcare Journal* 2021;8(1):e27-e31. doi: 10.7861/fhj.2020-0093

18. Clarke MA, Fruhling AL, Sitorius M, et al. Impact of Age on Patients' Communication and Technology Preferences in the Era of Meaningful Use: Mixed Methods Study. *J Med Internet Res* 2020;22(6):e13470. doi: 10.2196/13470 [published Online First: 20200601]

For peer review only

Remote consultations- Rheumatology

Interview schedule - patient

Thank you for agreeing to take part in this project. As you'll have read in the information document, we are interested in your experience and views of remote rheumatology consultations. So, in this call, I'll ask you some questions about these consultations – there are no right or wrong answers, we're just interested in your opinions. I'll record what is said in this call so that it can be typed up later, but any information that could identify you, such as names of people or places, will be removed. If at any time during the call you would like to stop, just let me know, and you do not have to answer any questions that you do not feel comfortable with.

Before we begin, do you have any questions?

[Start recorder]

To start with, can you tell me how many remote (telephone) rheumatology consultations you have had (roughly)?

And when did the remote consultations start? How long had you been having rheumatology appointments before the COVID pandemic?

Can you describe for me what typically happened when you attended the clinic for face-to-face consultations?

How does the session begin? Do you do any preparation before the session?

What information do you try to give the clinician?

How does the session end?

Do you feel the length of the sessions was appropriate (initial session = 30 mins, FU = 15 mins)

Can you describe for me what typically happens in your remote consultations?

How does the session begin? Do you do any preparation before the session?

What information do you try to give the clinician?

How does the session end?

What are the most important differences for you?

Did you receive a clinic letter informing you of your remote consultation?

Do you tend to read everything in your clinic letters or focus on, say, the appointment time?

What, if anything, have you found difficult in conducting remote consultations?

Expressing what is wrong?

Understanding instructions/advice?

Building rapport with clinician?

For peer review only

Technology difficulties – poor lines, hard to take videos of problem area etc.

Has anything helped you to overcome these difficulties?

For example, has asking certain questions helped you to gain a better understanding of what the clinician needs to know/your condition?

Support from partner/friend during the call?

Has anything about the remote consultation been better for you than when meeting in person?

Convenience? More relaxed at home? Not having to take time off work? No travel/parking costs? No waiting at the hospital?

Have you had any face-to-face consultations recently/since having remote consultations?

If yes, how did you find these?

Would you have any concerns if the clinic were to keep running consultations remotely (beyond COVID), at least some of the time?

For your level of care or safety?

Are some types of consultations better conducted remotely than others?

If the clinic was to start using video call, rather than telephone, for remote consultations, how would you feel about this?

If there are times when your remote consultation works particularly well, what do you think made the difference?

And what sorts of things make you feel a consultation hasn't gone very well?

Are there any situations where you would prefer a remote consultation?

For example, drug education, disease education, medication review.

Is there any support or training, for clinicians, other staff or for you, that you think would improve remote consultations?

For example, access to more advanced technology, guidance for clinicians on effective consultations?

That's all my questions, is there anything else you would like to add?

Thank them for their time and stop recorder.

For peer review only

Standards for Reporting Qualitative Research - checklist

SRQR item	Manuscript page number (line number, if applicable)
Item 1. Title	1 (1-2)
Item 2. Abstract	2 (1-23)
Item 3. Problem Formulation	5 (6-8)
Item 4. Purpose or research question	5 (8-13)
Item 5. Qualitative approach and research paradigm	5(8-9)
Item 6. Researcher characteristics and reflexivity	7 (4-8)
Item 7. Context: Setting/site and salient contextual factors; rationale	5 (10-11) and 6 (19-24)
Item 8. Sampling strategy	6 (8-17)
Item 9. Ethical issues pertaining to human subjects	6(4-6, 17, 20)
Item 10. Data collection methods	5 (16-17) and 6 (19-22)
Item 11. Data collection instruments and technologies	5 (20-24) and 6 (1-4), plus Supplementary file 1
Item 12. Units of study	7 (14-23)
Item 13. Data processing	7 (2-4)
Item 14. Data analysis	7 (4-12)
Item 15. Techniques to enhance trustworthiness	7 (4-12)
Item 16. Synthesis and interpretation	8 - 16
Item 17. Links to empirical data	Throughout Discussion, pages 16 - 19
Item 18. Integration with prior work, implications, transferability, and contribution(s) to the field	Throughout Discussion, pages 16 - 19
Item 19. Limitations	18 (20-24) and 19 (1-10)
Item 20. Conflicts of interest	None to declare, 20 (13)
Item 21. Funding	20 (15)

For peer review only

BMJ Open

Patient-Clinician Dynamics in Remote Consultations: A Qualitative Study of Cardiology and Rheumatology Outpatient Clinics in the UK

Journal:	*BMJ Open*
Manuscript ID	bmjopen-2022-070923.R1
Article Type:	Original research
Date Submitted by the Author:	24-Apr-2023
Complete List of Authors:	Grey, Elisabeth; University of Bath, Department for Health; University of Bristol Medical School<br>Brown, Frankie; University of Bath, Department for Health<br>Smith, Paula; University of Bath - Claverton Down Campus, Department of Psychology<br>Springett, Daniella; University of Bath, Department for Health<br>Augustine, Dan; Royal United Hospitals Bath NHS Foundation Trust; Royal United Hospitals Bath NHS Foundation Trust<br>Sengupta, Raj; The Royal National Hospital for Rheumatic Diseases, Bath<br>Peacock, Oliver; University of Bath, Department for Health<br>Gillison, Fiona; University of Bath, Department for Health
**Primary Subject Heading**:	Qualitative research
Secondary Subject Heading:	Health services research, Cardiovascular medicine, Rheumatology
Keywords:	Quality in health care < HEALTH SERVICES ADMINISTRATION & MANAGEMENT, QUALITATIVE RESEARCH, RHEUMATOLOGY, CARDIOLOGY

SCHOLARONE™ Manuscripts

I, the Submitting Author has the right to grant and does grant on behalf of all authors of the Work (as defined in the below author licence), an exclusive licence and/or a non-exclusive licence for contributions from authors who are: i) UK Crown employees; ii) where BMJ has agreed a CC-BY licence shall apply, and/or iii) in accordance with the terms applicable for US Federal Government officers or employees acting as part of their official duties; on a worldwide, perpetual, irrevocable, royalty-free basis to BMJ Publishing Group Ltd ("BMJ") its licensees and where the relevant Journal is co-owned by BMJ to the co-owners of the Journal, to publish the Work in this journal and any other BMJ products and to exploit all rights, as set out in our licence.

The Submitting Author accepts and understands that any supply made under these terms is made by BMJ to the Submitting Author unless you are acting as an employee on behalf of your employer or a postgraduate student of an affiliated institution which is paying any applicable article publishing charge ("APC") for Open Access articles. Where the Submitting Author wishes to make the Work available on an Open Access basis (and intends to pay the relevant APC), the terms of reuse of such Open Access shall be governed by a Creative Commons licence – details of these licences and which Creative Commons licence will apply to this Work are set out in our licence referred to above.

Other than as permitted in any relevant BMJ Author's Self Archiving Policies, I confirm this Work has not been accepted for publication elsewhere, is not being considered for publication elsewhere and does not duplicate material already published. I confirm all authors consent to publication of this Work and authorise the granting of this licence.

For peer review only

Patient-Clinician Dynamics in Remote Consultations: A Qualitative Study of Cardiology and Rheumatology Outpatient Clinics in the UK

Elisabeth Grey[a.b]*, Frankie Brown[b], Paula Smith[c], Daniella Springett[b], Dan Augustine[b, d], Raj Sengupta[e, f], Oliver Peacock[b] and Fiona Gillison[b]

[a] Bristol Medical School (Population Health Sciences), University of Bristol, Bristol, UK

[b] Department for Health, University of Bath, Bath, UK

[c] Department of Psychology, University of Bath, Bath, UK

[d] Royal United Hospitals Bath NHS Foundation Trust, Bath, UK

[e] Royal National Hospital for Rheumatic Diseases, Bath, UK

[f] Department of Pharmacy and Pharmacology, University of Bath, Bath, UK

*Corresponding author email: e.b.grey@bath.ac.uk

Word count: 4763

For peer review only

For peer review only - http://bmjopen.bmj.com/site/about/guidelines.xhtml

1

For peer review only

Abstract

Objective: Explore the experiences of patients and clinicians in rheumatology and cardiology outpatient clinics during the first year of the COVID-19 pandemic, focusing on the impact of remote consultations on interpersonal dynamics.

Design: Qualitative study using semi-structured interviews, conducted between February and June 2021.

Setting: The rheumatology and cardiology departments of a general hospital in England, UK.

Participants: All clinicians and a convenience sample of 100 patients in each department who had taken part in a remote consultation in the past month were invited to take part. Twenty-five interviews were conducted (13 with patients, 12 with clinicians).

Results: Three themes were developed through the analysis: adapting to the dynamics of remote consultations, impact on the patient's experience, and impact on the clinician's experience. The majority of remote consultations experienced by both patients and clinicians had been via telephone. Both clinicians and patients found remote consultations to be more business-like and focused, with the absence of pauses restricting time for reflection. For patients with stable, well-managed conditions, remote consultations were felt to be appropriate and could be more convenient than in-person consultations. However, the loss of visual cues meant some patients felt they could not give a holistic view of their condition and limited clinicians' ability to gather and convey information. Clinicians adjusted their approach by asking more questions, checking understanding more frequently, and expressing empathy verbally, but felt patients still shared fewer concerns remotely than in person; a perception with which patients concurred.

Conclusions: These findings highlight the importance of ensuring, for each patient, that remote care is appropriate. Future research should focus on developing ways to support both clinicians and

For peer review only - http://bmjopen.bmj.com/site/about/guidelines.xhtml

patients to gather and provide all information necessary during remote consultations, to enhance communication and trust.

Article summary

Strengths and limitations of this study

- A key strength of this study is considering clinicians' experiences of remote consultations alongside patients', enabling the commonalities and differences between these perspectives to be seen.
- Participants were recruited from two departments of one general hospital - the findings may not be representative of other hospitals.
- This study focused specifically on experiences during the first year of the COVID-19 pandemic – acceptance and expectations for remote consultations is likely to evolve over time as people become more familiar with this format and technology advances.

Keywords: remote consultations; patient experience; clinician experience; interpersonal communication

For peer review only

For peer review only - http://bmjopen.bmj.com/site/about/guidelines.xhtml

Introduction

The COVID-19 pandemic precipitated the rapid implementation of remote consultations in both primary and secondary care [1 2] and remote delivery of health services is set to continue. In the UK, for example, NHS planning guidance for 2022 sets out the ambition that "at least 25% [of outpatient appointments] should be delivered remotely by telephone or video consultation (equivalent to c.40% of outpatient appointments that don't involve a procedure)" [3]. Research conducted prior to the COVID-19 pandemic can inform us on user experience when planned remote services are implemented, but the rapid rise in the use of technology during the pandemic means that the background level of skill and acceptability has changed[4]. Recent research has pointed to a need for the post-pandemic remote delivery model to evolve in both primary [5] and secondary care [6] to reflect the variation in and changing levels of patients' and clinicians' confidence and skills, as well as changes in technology.

The focus of this article is remote consultations conducted in secondary care (specifically cardiology and rheumatology outpatient appointments). Remote consultations have been used and studied more extensively in primary care [5 7] and, while much of this research will be relevant to secondary care settings, there is a need to identify the unique challenges and benefits of remote consultations in secondary care too. Research has highlighted some of the advantages and disadvantages of remote consultations for outpatient care during the COVID-19 pandemic. Routinely collected feedback reported advantages, such as reduced stress, enhanced accessibility, cost, and time savings [6], and disadvantages including technical difficulties [5 6 8] and the inability to conduct physical examination [9-11]. Remote consultations are also reported to challenge communication due to the lack of visual cues, such as body language and facial expressions, in telephone consultations (e.g., to gauge patient understanding) [4 9 12] and reduced cues (e.g., not able to see the patients' movement in

For peer review only - http://bmjopen.bmj.com/site/about/guidelines.xhtml

and out of a consultation room) and the need to support patients' autonomy while ensuring self-examinations are conducted correctly in the case of videocalls [4 13].

Relatively limited qualitative research is available exploring some of these difficulties in more depth. Analysis of three studies incorporating interviews with 35 patients with musculoskeletal conditions found acceptability of the consultation format varied independently of characteristics of the condition alone [4]. Clinicians have reported barriers such as a lack of confidence in patients' ability to communicate remotely and patients endorsed clinicians' views that remote consultations are not as satisfactory when building new relationships [4]. Gilbert and colleagues (2022) reported how interpersonal relations influence the acceptability of video consultations in orthopaedics, suggesting the norms and expectations for remote consultations of both staff and patients play an important role in determining satisfaction with a consultation.

As remote consultations are set to remain a part of health service delivery, it is important to understand how they impact patient-clinician dynamics (that is, the interaction between patient and clinician) and identify ways to ensure they fully meet the needs of both. In this qualitative interview study, we explored patients' and clinicians' experiences of remote consultations conducted during the first year of the COVID-19 pandemic from two outpatient settings in a hospital in south west England, UK, that deliver care for people with chronic conditions: rheumatology and cardiology. This project came about as a collaboration between a University and local hospital interested in exploring how to learn from and improve their delivery of care via telephone and digital technology for the longer term, following initial modes of implementation during the COVID-19 pandemic. Therefore, in this qualitative exploration our objectives were to understand how patients and clinicians experienced remote consultations and identify factors that facilitated and hindered the provision of remote outpatient care.

For peer review only - http://bmjopen.bmj.com/site/about/guidelines.xhtml

Methods

Design

Data were gathered using in-depth semi-structured interviews conducted by telephone or online call between February and June 2021. The interview topic guide was developed by the research team, including collaborating clinicians, and informed by a rapid review of published literature on the acceptability, advantages and disadvantages of delivering remote consultations in secondary care (conducted on published research up to February 2021). The interview schedules comprised open-ended questions and prompts to elicit detailed responses, with questions focusing on participants' experiences of face-to-face and remote consultations, markers of good quality remote consultations, and barriers and facilitators to successful remote consultations. Separate interview schedules were developed for patients and staff, and for rheumatology and cardiology, to ensure the question wording was appropriate for participants' experience. Example interview schedules for rheumatology patients and clinicians are provided in Supplementary file 1. This study received ethical approval from the Research Ethics Approval Committee for Health at the University of Bath (Reference Number: EP 20/21 008). No personal data was transferred from the hospital to the research team, and only anonymised interview transcripts were retained after the study, stored securely in accordance with UK data protection regulations.

Setting

The hospital is a major provider of acute and specialist services in the south west of England. It draws patients from a wide range of socio-economic backgrounds as well as both rural and urban contexts, but with little ethnic diversity. For this research, departments providing remote outpatient care were invited to take part – four departments responded: Dermatology, Cardiology, Older people's and Rheumatology. Work with the Dermatology department took a slightly different focus

For peer review only

For peer review only - http://bmjopen.bmj.com/site/about/guidelines.xhtml

as this team were using a specific software platform. Work with the Older people's unit followed a different analysis and is reported elsewhere[14].

Participants and Recruitment

Clinicians: Emails were sent to all clinicians in patient-facing job roles working in participating departments, inviting them to take part in the study and providing a link to an online information sheet and consent form. The invitation came from clinical colleagues (authors DA and RS, who work at the hospital and collaborated on this project with the other authors, based at the university) but it was made clear that the interviews and analysis would be conducted independently by the research team and participation would be anonymous.

Patients: Patients who had completed at least one remote consultation in participating departments in the past month were eligible to take part. A sample of 100 eligible patients in each of the two departments was identified from patient records and invitation letters and information sheets were sent to them in the post; invitations directed patients to either complete an online consent form or contact the research team directly if they were interested in taking part. To boost recruitment in cardiology, the researcher spent time in the outpatient clinic to explain the study to clinicians and patients to encourage recruitment. We anticipated a response rate of about 5-10% from each department; the invitation sample of 100 from each department therefore reflects an effort to recruit at least ten patients.

Procedure

Participants first completed an online form to provide consent and then demographic information including age group, gender, ethnic group, length of time receiving treatment from the department (patients), and professional role and length of time in profession (clinicians). For some patients, consent and demographic data were collected at the start of the interview calls. Semi-structured

For peer review only - http://bmjopen.bmj.com/site/about/guidelines.xhtml

interviews were conducted remotely, either by telephone or video call, by two researchers (EG and DS).

Patient and public involvement

Due to the lockdown restrictions in place at the time of study planning and the short time limit in which to complete the study for the funders, we were unable to involve patients and the public in the design and conduct of the study. Findings were, however, reported to patient involvement groups at the hospital and their reflections on these helped to inform a further study.

Analysis

Interviews were audio recorded and transcribed intelligent verbatim. Personal, identifying information was removed from transcripts prior to analysis. Transcripts were analysed thematically [15 16] using NVivo version 12 software to help organise, code and explore the data. EG and PS (first read a subset of the transcripts to familiarise themselves with the data; EG then coded the subset and compiled an initial coding framework, in discussion with PS. EG and DS (a doctoral student, supervised in analysis by EG) then coded the remainder of the transcripts adding new codes where necessary, continuing to discuss and refine the framework with PS. EG organised the final codes into themes which were discussed and refined with the research team, trying to ensure each theme was distinct and well supported by individual transcripts as well as the dataset as a whole. We took a critical realist perspective, believing that reality exists, independent of observers, but can only be understood through individuals' perceptions and interpretations [17]. We followed an inductive approach to generating codes and themes in the initial analysis, but the selection and development of final themes was driven by the aim of focusing on the psychosocial aspects of consultations. EG and PS are both experienced qualitative researchers with a background in health psychology, DS is a doctoral student who was supervised in the analysis by EG; none work in the hospital. PS was formerly a nurse but did not have experience of remote consultations as either clinician or patient;

For peer review only - http://bmjopen.bmj.com/site/about/guidelines.xhtml

EG and DS are not clinicians and did not have experience of remote consultations as patients. We have followed the Standards for Reporting Qualitative Research (SRQR) and the completed checklist can be found in Supplementary file 2.

Results

In total, 25 people responded to the invitations and were interviewed: 12 clinicians (six cardiology and six rheumatology) and 13 patients (three cardiology and ten rheumatology). Due to the low numbers involved, overall demographic data for the sample are reported here to protect clinicians' and patients' identities. The clinician participants from each department included both nurses (N = 5) and doctors (N = 7), seven were male and the majority (75%) were white, with 6 to 46 years of experience in the profession. All patients were white, the majority (67%) were female and ranged in age group from 35-44 years to over 75 years. Patients had been receiving treatment from their respective departments for between two months and over 20 years. Most remote consultations conducted in this hospital were by telephone, although a few cardiology clinicians and two patients had also conducted video-call consultations; the results discussed here, thus, mostly relate to telephone consultations except where videocalls are explicitly mentioned. All remote consultations discussed were follow-up appointments as initial consultations were conducted in-person in the hospital. Prior to the pandemic, all consultations (initial and follow-up) took place in-person at the hospital.

Clinicians and patients alike felt the dynamics of remote consultations would be suitable for regular, routine follow-up appointments where the patient's condition was stable; they were considered unsuitable for initial appointments and breaking bad news. Remote consultations were thought to offer advantages in terms of convenience and improving access for patients who live far away from the hospital, who struggle to get time off work for an appointment or who struggle to afford the

For peer review only - http://bmjopen.bmj.com/site/about/guidelines.xhtml

travel costs. Three themes were developed that went beyond this feedback on the practicalities of remote consultations to explore the interpersonal effects of moving to a remote model of care; the key points for each theme are summarised in Table 1. The first, largest theme, which was consistent across both patient and clinician participants, was *Adapting to the dynamics of remote consultations*. Theme two focused on the *Impact on the patient's experience* of remote consultations and the final theme was on the *Impact on the clinician's experience.* The same topics and perspectives were raised by interviewees from both departments, so are presented together within each theme.

Table 1. Summary of key points within each theme

Theme	Key points
Adapting to the dynamics of remote consultations	Remote consultations tend to be more focused, "business-like" - this can make it harder to build a rapport and mean patients do not raise all their concerns<br>The lack of visual cues in telephone consultations can mean it takes longer to gather/convey information<br>Relying on oral communication to convey empathy, reassurance, encouragement etc. is not always satisfactory<br>The lack of visual cues in telephone consultations makes it harder to assess patients' understanding
Impact on the patient's experience	The focused style and lack of visual information leaves some patients feeling unable to convey a holistic view of themselves<br>The physical presence of a clinician can be reassuring; the lack of it can leave patients feeling less well cared for and uncertain of their clinician's level of concern
Impact on the clinician's experience	Relying on only oral communication to gather and convey information can be exhausting<br>In the majority of cases clinicians are confident of their ability to accurately assess and treat patients remotely, but there is a heightened fear of missing important signs when not seeing patients face-to-face<br>The diminished social contact of remote consultations may be less satisfying to clinicians

For peer review only - http://bmjopen.bmj.com/site/about/guidelines.xhtml

Theme 1: Adapting to the dynamics of remote consultations

Clinicians and patients both reported that remote consultations tended to be more focused, with less talking about matters not directly related to the patient's condition.

> *Patients don't tend to chat as much either interestingly. People I know who are chatty, and I have to keep on pulling them on track when I'm face to face with them, are much more focused on just what they need to tell me. The actual interview tends to take less time, because they don't side track themselves so much when they're on the phone. (RC03, rheumatology nurse, female)*

This was attributed by some to the different conventions associated with telephone calls compared to those of meeting people in person, with telephone calls being more typically used for short or functional exchanges of information.

> *They seem to have been briefer and quicker because we both know the difficulties. I'm kind of used to dealing with technology so I'm used to the way you use it, you talk, they talk … it's a very precise form of communication. (RP12, rheumatology patient, male)*

There was some indication that in face-to-face appointments this 'chat' provides a function of establishing rapport and trust and may also contribute to setting a more relaxed tone in which patients feel comfortable raising issues that they worry are less important (explored further in Theme 2).

> *There's something about the phone that means that I'm also more business-like and think 'oh no, I won't waste time asking that', and maybe brush over things a bit more. Whereas if I was sitting in a consultation and actually looking at the consultant, I might open up a little bit more. (CP06, cardiology patient, female)*

For peer review only - http://bmjopen.bmj.com/site/about/guidelines.xhtml

While this more focused dynamic could work well for patients whose conditions were stable and who had an established relationship with the clinicians, it made it harder to develop a rapport when the clinician and patient had not previously met.

> *I would never ever want to meet a person first time over the phone. I think it's always good to look somebody in the eye properly over a table rather than virtually. And then it's a lot easier to have problem phone calls afterwards because you've met them, you know them. You get a sense for somebody, I think. (CP07, cardiology patient, male)*

Having to make calls from unknown numbers was also felt to present a barrier to establishing trust – as one doctor highlighted, this required patients to accept that they were not hoax callers.

> *I think on a telephone consult it's quite difficult to get across who you are... because our numbers are withheld from the hospital you're taking it for granted that they trust you. Whereas if you've got somebody in a room you build that rapport immediately, I think, by introducing yourself and having eye contact and that kind of thing. (RC01, rheumatology doctor, male)*

While remote consultations were perceived to be more focused, clinicians often reported taking longer to question patients as they needed to find new ways of gathering the information needed for treatment decisions that would previously have been evident through visual cues.

> *Obviously, you can tell whether somebody looks well or unwell when they walk in... Whereas obviously I don't know that on the telephone, so I have to ask them. You can tell that a bit from the way they talk and what they're talking about, and the energy in their voice, or the lack of energy in their voice. So yeah, there is a little bit of that that you don't see, so you have to ask more questions. (CC04, cardiology doctor, male)*

For peer review only - http://bmjopen.bmj.com/site/about/guidelines.xhtml

Clinicians discussed how they tried to create space for the discussion of additional questions or concerns in remote consultations, just as they would in face-to-face consultations, to try and encourage greater communication when consulting remotely. They also explained how they tried to use their own body language in face-to-face consultations to express empathy, reassurance or encouragement. While they found means of expressing these emotions orally in remote consultations, this was not always felt to be effective, particularly for 'stoical' patients (i.e., those who are reluctant to express that they are in pain).

> *Because you can use your own body language to actually encourage them to talk, which is really hard to do over the phone; there's only so many ways you can rephrase things until they get fed up with you. (RC03, rheumatology nurse, female)*

> *Sometimes they're not prepared to talk to you over the phone about it. Particularly if you don't know them very well, some people are still reluctant to tell you everything… Some of them are very stoical, our patients. (RC03, rheumatology nurse, female)*

The missing visual cues in remote consultations also made it harder for clinicians to assess patients' understanding of the information and advice discussed, again leading them to ask more questions.

> *I find that I tend to repeat myself quite a lot to check if I'm getting through to them, because I can't assess nodding or smiles or visual clues to know that I'm making sense. So, I constantly say "I hope that's clear", "I hope that makes sense to you". So, it's a bit longwinded I find, some of my consultations, because I keep checking that they've understood what I'm trying to say. (CC01, cardiology doctor, male)*

Clinicians also relayed how visual cues from the patient in face-to-face consultations were relied upon for determining subtler aspects of their (i.e., the clinician's) conduct, such as how to pitch the level of information or their tone of voice.

For peer review only - http://bmjopen.bmj.com/site/about/guidelines.xhtml

The way they look at you, whether they look in your eyes or whether they don't look at you at all. That can reflect them being scared a lot of the time, or maybe not understanding. (CC04, cardiology doctor, male)

You can tell how somebody feels by looking at their face and sometimes you can tell if there's something else going on that they want to talk about by looking at them and seeing how they are. You can also tell if somebody's very happy with how they are being treated by their body language. That's been one of the challenges that I've found with telephone consults, is knowing how somebody is just by being around them potentially. (RC01, rheumatology doctor, male)

Despite clinicians tending to ask more questions in remote consultations, the focused dynamic meant there was also a tendency not to leave pauses. Breaks in conversation during consultations are important for allowing patients time to think or to process bad news together and the lack of pauses in remote consultations was felt by both patients and clinicians.

I think it's hard because in a face-to-face consultation you can have pauses, where you're thinking about things… It feels less pressured. Telephone conversations are often 'I talk and then you talk', but there's not much time for a pause or reflection of what has been said. And then it's over, and you can't. It's like often if you finish a consultation, you go to the door and you sort of say "Oh, what about this, can you just tell me quickly about this before I go?". Whereas with a phone call once it's over, it's over and that's it." (CP09, cardiology patient, female)

If you've just got time just to sit and accept the silence together that's sometimes part of it, and on a telephone you're always going to try and fill that awkward silence. Whereas if they're there with you, you don't have to, you can just let that go and then just wait. (CC04, cardiology doctor, male)

For peer review only - http://bmjopen.bmj.com/site/about/guidelines.xhtml

Theme 2: Impact on the patient's experience

When patients were not physically seen by clinicians, some felt that they couldn't convey a full, holistic view of themselves. Patients reported finding it hard to find the right words to discuss their condition, or they could not or did not want to talk about more indirect consequences of their condition or treatment such as weight gain or fatigue. In a face-to-face consultation, they felt the clinician would be able to see these without them needing to raise the topic first themselves

> *When you're not meeting with them, they're just on the phone, it doesn't feel like they're totally across it. Because they haven't seen you, they haven't examined you. They don't know what you look like… she could probably tell by looking whether I'm in a bad phase or a good phase or whether the bad phases are worse than they used to be. (RP08, rheumatology patient, female)*

> *I think their intuition does play a part in medicine, with doctors… there's nothing quite like sitting face-to-face seeing what colour the skin is, what the general feeling is, what the energy is like. (RP16, rheumatology patient, female)*

As one patient pointed out, this may be partially remedied by having video rather than telephone consultations.

> *Sometimes if it's [a clinician] I don't know they'll say, "do you manage to look after yourself?" … hopefully through a video they can see a bit of my home and how busy life is It gives the impression you feel they are on my wavelength a bit faster. That I don't have a carer coming in!" (RP09, rheumatology patient, female)*

The physical presence of a clinician in face-to-face consultations was a clear sign that someone was looking after them and also enabled patients to benefit from the visual cues in a clinician's body language (e.g., when expressing empathy or concern).

For peer review only - http://bmjopen.bmj.com/site/about/guidelines.xhtml

To see them and be reassured by their demeanour - because you can tell when you look at people whether they're concerned about something or not. They might reassure you verbally, but you can pick up if they think 'oh no this is something we need to investigate further'. (CP09, cardiology patient, female)

Thus, telephone consultations were felt by some to provide less reassurance and this seemed to be felt particularly when patients were suffering worse symptoms or experiencing more uncertainty.

It would be peace of mind as well, because if the consultant says "I don't think there's any need [for treatment]", at least I feel that they have seen my ailment… if it was me, personally, in pain, I really feel I need to see- I would want to see them face-to-face. (RP15, rheumatology patient, female)

As can be seen from the last quotation, physical presence provides reassurance not only through conveying visual cues of empathy but also through enabling patients to convey what they consider a more holistic picture of themselves, including what they may struggle to express in words.

Theme 3: Impact on the clinician's experience

Clinicians reported that the additional effort needed to gather information, develop rapport and convey interest and understanding solely through speech, without seeing patients, could be exhausting.

Telephone consults can be quite draining though because you end up talking quite a lot. So, I think if you're doing telephone consults non-stop day in, day out, then from a clinician point of view it would be quite draining. (RC01, rheumatology doctor, male)

When they have got tender swollen joints it's just really difficult - it's a long drawn out conversation to identify where the problems are if people have got multiple troublesome

For peer review only - http://bmjopen.bmj.com/site/about/guidelines.xhtml

joints. If it's just a couple that's not really a problem, but if they're having a lot of problems, I just find that a challenging conversation. (RC02, rheumatology nurse, female)

Without being able to see patients or conduct physical examinations, clinicians also reported feeling a fear that they might be missing important signs of disease or misdiagnosing patients. Although in the vast majority of cases clinicians were confident in their ability to accurately assess and treat patients remotely, there could nonetheless be a background-level doubt, particularly for 'stoical' patients.

There's always going to be that slight niggle at the back of your mind about the patient who thinks they are doing very well but actually they've got some gradual progressive disease that they are not aware of. (RC06, rheumatology doctor, male)

You will always have a clue, there will be blood tests and the letter ... But you do worry, that there might be someone that, because you haven't seen them, that you miss a diagnosis or you don't take on board the severity of a diagnosis. (CC02, cardiology doctor, male)

One clinician also highlighted the potential for safeguarding issues to arise when they couldn't see patients, again causing uncertainty for staff.

I have had a situation… calling somebody's mobile phone and their partner answering and saying that they were too unwell to come to the phone. And then that brings up like a safeguarding issue - is that patient at risk? If they're so unwell that they can't come to the phone… Are they actually unwell? (RC01, rheumatology doctor, male)

Not being able to see patients meant that clinicians missed an important source of positive feedback, such as patients smiling or acting more at ease following a successful course of treatment. This, in addition to the diminished rapport, made remote consultations less sociable for clinicians.

For peer review only - http://bmjopen.bmj.com/site/about/guidelines.xhtml

It's definitely, in some ways, less satisfying because you can't see the patient smile. And sometimes when patients are walking out the clinic room, you have a bit of banter... There isn't that additional kind of interaction which you get from a face-to-face consult. (CC01, cardiology doctor, male)

I do think we are visual. I think we do relate to people better when we can see them… I prefer that myself, rather than over the phone. (CC05, cardiology nurse, female)

Overall, the additional sources of uncertainty and diminished rapport and sociability of remote consultations seemed to make them less satisfying for clinicians and potentially more draining. However, the clinicians also acknowledged the practical advantages of remote consultations and some highlighted that with time and experience, conducting remote consultations became easier.

Discussion

This paper reports on the interpersonal impacts of remote consultations for clinicians and patients in the year following the onset of the COVID-19 pandemic. Both clinicians and patients perceived a shift in the tone of consultations to be more "business-like" and focused when delivered remotely rather than in person, attributing this to the conventions of telephone calls. Both patients and clinicians flagged the absence of pauses in conversations, removing time for reflection on information delivered, particularly when this was bad or unwelcome news. Clinicians reported having adjusted their approach by asking more questions, checking understanding more frequently and finding ways to express their own understanding and empathy verbally where they would previously have relied on body language. Some found this more tiring than face-to-face consultations. Even with such adaptations, there was acceptance among clinicians that patients seemed less comfortable discussing certain topics and disclosing as much information as they would have done in person; this may be a particular concern among 'stoic' patients. Supporting clinicians'

For peer review only - http://bmjopen.bmj.com/site/about/guidelines.xhtml

For peer review only

perceptions, patients reported being less likely to raise 'small' issues in remote consultations that they would have done in face-to-face consultations and felt that they had been less able to communicate a holistic version of themselves.

The present study adds to the small body of qualitative research that incorporates both patient and health care professional perspectives, which provides an opportunity to explore reciprocal or interacting effects. A qualitative study of patients with musculoskeletal conditions identified that, while remote consultations were suitable and convenient for patients with stable conditions, face-to-face consultations enabled superior communication that was particularly sought when patients were in more distress or pain [4]. These findings were echoed by the patients in our sample, who reported that they would want to be seen in person when their symptoms were bad. Among our sample of cardiology and rheumatology clinicians, we found concerns that patients were not as willing or able to communicate as fully in remote consultations as when face-to-face and an awareness that they do not get a comprehensive picture without seeing patients in person, leaving them uncertain as to whether there is more they should be doing for their patients. Some of the patients and clinicians in our sample speculated that these difficulties may partly be overcome with the use of video call technology (rather than the telephone calls that were employed in most remote consultations). Indeed a survey study of patient and staff perceptions of video consultations conducted from an older adult outpatient unit in the UK found high levels of satisfaction with this format, particularly among patients [6]. In contrast, however, an interview study with patients and clinicians at an orthopaedic centre in the UK found some clinicians perceived video call assessments to be less accurate and more likely to result in worsening of a patients' condition; this corresponded to some patients' experiencing slower rehabilitation and perceiving remote appointments to be less effective than face-to-face [18]. This may help to explain, in part, the results of a mixed methods study conducted in a UK outpatient department following the rapid implementation of a remote video consultation platform which indicated that, while all patients reported that the consultations met

For peer review only - http://bmjopen.bmj.com/site/about/guidelines.xhtml

their needs, they were less satisfied than clinicians that they had communicated everything they wanted to (86% vs 95%) [19]. A quarter of clinicians showed some recognition of this, reporting a belief that patient experience of a remote video consultation was worse than that in a face-to-face clinic [18 19]. This echoes findings from primary care, where general dissatisfaction with video consultations among both patients and practitioners has been reported, emphasising a belief that the best therapeutic care is delivered in person [7]. Thus, research suggests that patients and clinicians, when communicating remotely, can feel a lack of certainty that either they or the other party has read the situation correctly; this can contribute to perceiving remote consultations to be poorer quality than traditional face-to-face consultations.

Implications

The present study has confirmed that while remote consultations are acceptable and can provide a basis for care, whether delivered in addition to or as an alternative to usual care[20], they do not provide a like-for-like exchange [21]. Past work has flagged the need to match the format of consultations to different types of appointment (e.g., remote consultations are less appropriate for initial appointments and breaking bad news, but may work well for the follow-up of stable cases [22]), but there may also be benefit in matching the format to types of patient (e.g., stoic, health anxious, higher risk) and, potentially, to preferences among patients and clinicians [4]. Practical means of identifying how to enable flexible delivery in responding to these variations when implementing at scale may be essential for the successful incorporation of remote consultations into future mainstream care.

Given that some degree of remote care is certain to continue [21], further work is needed to find effective ways of supporting patients to give, and clinicians to gather, all necessary information during remote consultations. This could help to set the expectations for and, in turn, satisfaction with remote consultations of both patients and clinicians [18]. This may involve, for example,

For peer review only - http://bmjopen.bmj.com/site/about/guidelines.xhtml

additional training for clinicians in communication techniques for remote consultations, different appointment structures to allow space for reflection, and exploring other approaches that could help to improve both patient and professional confidence and satisfaction. Similarly, educational resources could be developed for patients to provide advice on how they can best prepare and encourage them to mention both major and minor issues and to use their full allocated time slot rather than stick to usual "business-like" norms for phone contact.

As both patients and clinicians become more familiar with remote consultations, long-term research will be useful in identifying changes (or the lack of) in the impact of technological restrictions, comfort with communicating via telephone or video call, clinician job satisfaction and other unanticipated consequences of greater remote provision. While patients were generally happy to accept remote care delivered via telephone during the peaks of the pandemic, seeing it as preferable to no care or to increasing their risk of infection by visiting the hospital, this may have changed now that everyday life is increasingly less restricted. Furthermore, the increasing rollout of remote consultations has important implications in terms of health inequalities, with a greater reliance on technology potentially exacerbating existing disparities between age, ethnic and economic groups as well as for those with disabilities [4]. The small sample of the present study prevents us from comparing different patient groups, however, even within our sample there was evidence of some patients being less confident in using digital technology to access health care. While providing information and support to improve digital literacy may help to limit increasing health inequality, the importance of cultural group norms and individual preferences should not be overlooked. By pushing for more consultations to be held remotely, there is a danger that patients who are unable to access or unconfident in using telephones or video call technology may feel they are burdening the system in requesting a face-to-face appointment – this in turn could lead to them not seeking help when they need it, further increasing health inequalities. It is thus important not only for face-

For peer review only - http://bmjopen.bmj.com/site/about/guidelines.xhtml

to-face consultations to always be available but that they are easily accessible and that patients are made to feel welcome to request such an appointment.

Limitations

The participants in the present study are from a limited pool of respondents from a regional hospital in south west England. Unfortunately, we did not collect data on participants' cultural background or preferred languages, factors which are likely to influence individuals' access to and experience of health care [23]. It should also be noted that our sample did not include young adults (<35 years), who are likely to have different acceptance levels and expectations for remote health communication [24]. Therefore, a wider range of considerations and influences may have been discussed if conducted in hospitals based in other communities. While we invited 100 patients from both departments, this was much more successful with rheumatology patients, so these are more strongly represented. Unfortunately the funding for this research was time limited and so we were not able to conduct any additional recruitment drives to increase or achieve parity in the sample. Most remote consultations within the hospital were conducted via telephone. This is not unusual across the NHS, due to both the availability of equipment and private space for clinicians, as well as concerns about patient access to technology and digital skills. Video consultations are likely to have a different dynamic but were experienced by only two patients in this study. Finally, these interviews took place during a lockdown period and before vaccines were available free of charge for all adults. As such, acceptance of the need to attend remotely was likely higher among both patients and clinicians than when concerns about the risk of infection are lower.

Conclusions

The use of remote consultations in secondary outpatient care is largely accepted by patients and clinicians, and set to remain in future healthcare delivery. While technical difficulties in the delivery

For peer review only - http://bmjopen.bmj.com/site/about/guidelines.xhtml

of remote consultations are rapidly being overcome, more attention is needed on identifying ways to ensure remote consultations are employed appropriately and that, when they are, patients and clinicians are best enabled to communicate effectively. Remote consultations may never be able to fully replicate the interpersonal environment of face-to-face consultations, but research can help to identify ways to ensure that important elements of consulting are not lost and may also highlight traits of remote communication that are particularly suited to certain patients or clinicians.

Author contributions

EG, FB, FG and PS were responsible for the study conception and design. EG and DS conducted the interviews and coded the transcripts; EG was responsible for developing the coding framework and thematic analysis with input from PS. The results and interpretation were discussed among all authors (EG, FB, PS, DS, DA, RS, OP and FG). EG composed a first draft of the manuscript with contributions from FB and FG. All authors commented on the draft manuscript. All authors read and approved the final manuscript.

Acknowledgements

We would like to thank all patients and clinicians who participated in this study. We are also grateful to the health professionals who provided feedback on the findings in departmental workshops.

Ethics approval and consent to participate

The protocol was approved by the Research Ethics Approval Committee for Health at the University of Bath (Reference Number: EP 20/21 008). All methods were carried out in accordance with the relevant guidelines and regulations. Informed consent was obtained and documented electronically from all participants prior to each interview.

Competing interests

For peer review only

For peer review only - http://bmjopen.bmj.com/site/about/guidelines.xhtml

None to declare.

Funding

This work was supported by the UKRI Strategic Priorities Fund: Evidence Based Policy Making theme.

Availability of data and materials

The datasets generated and analysed in this study are not publicly available to reduce the risk of breaking the confidentiality of the study participants, but are available from the corresponding author on reasonable request.

For peer review only

For peer review only - http://bmjopen.bmj.com/site/about/guidelines.xhtml

References

1. Gilbert AW, Billany JCT, Adam R, et al. Rapid implementation of virtual clinics due to COVID-19: report and early evaluation of a quality improvement initiative. *BMJ Open Qual* 2020;9(2) doi: 10.1136/bmjoq-2020-000985

2. Silva AB, Sindico SRF, Carneiro AC, et al. COVID-19 Remote Consultation Services and Population in Health Inequity-Concentrating Territories: A Scoping Review. *Telemedicine and e-Health* 2021;27(8):881-97. doi: 10.1089/tmj.2021.0145

3. NHS England. 2021/22 priorities and operational planning guidance: October 2021 to March 2022. https://www.england.nhs.uk/wp-content/uploads/2021/09/C1400-2122-priorites-and-operational-planning-guidance-oct21-march21.pdf, 2021.

4. Paskins Z, Bullock L, Manning F, et al. Acceptability of, and preferences for, remote consulting during COVID-19 among older patients with two common long-term musculoskeletal conditions: findings from three qualitative studies and recommendations for practice. *BMC Musculoskeletal Disorders* 2022;23(1):312. doi: 10.1186/s12891-022-05273-1

5. Murphy M, Scott LJ, Salisbury C, et al. Implementation of remote consulting in UK primary care following the COVID-19 pandemic: a mixed-methods longitudinal study. *Br J Gen Pract* 2021;71(704):e166-e77. doi: 10.3399/BJGP.2020.0948 [published Online First: 20210225]

6. Bradwell H, Baines R, Edwards KJ, et al. Exploring Patient and Staff Experiences With Video Consultations During COVID-19 in an English Outpatient Care Setting: Secondary Data Analysis of Routinely Collected Feedback Data. *JMIR Form Res* 2022;6(3):e30486. doi: 10.2196/30486 [published Online First: 21.3.2022]

For peer review only - http://bmjopen.bmj.com/site/about/guidelines.xhtml

7. Greenhalgh T, Ladds E, Hughes G, et al. Why do GPs rarely do video consultations? qualitative study in UK general practice. *British Journal of General Practice* 2022;72(718):e351. doi: 10.3399/BJGP.2021.0658

8. Gilbert AW, Mentzakis E, May CR, et al. Patient preferences for use of virtual consultations in an orthopaedic rehabilitation setting: Results from a discrete choice experiment. *J Health Serv Res Policy* 2021:13558196211035427. doi: 10.1177/13558196211035427 [published Online First: 2021/08/03]

9. Isautier JM, Copp T, Ayre J, et al. People's Experiences and Satisfaction With Telehealth During the COVID-19 Pandemic in Australia: Cross-Sectional Survey Study. *J Med Internet Res* 2020;22(12):e24531. doi: 10.2196/24531 [published Online First: 20201210]

10. Jones MT, Arif R, Rai A. Patient Experiences With Telemedicine in a National Health Service Rheumatology Outpatient Department During Coronavirus Disease-19. *J Patient Exp* 2021;8:23743735211034973.

11. Sloan M, Lever E, Harwood R, et al. Telemedicine in rheumatology: A mixed methods study exploring acceptability, preferences and experiences among patients and clinicians. *Rheumatology (Oxford)* 2021 doi: 10.1093/rheumatology/keab796 [published Online First: 20211026]

12. Liberati E, Richards N, Parker J, et al. Remote care for mental health: qualitative study with service users, carers and staff during the COVID-19 pandemic. *BMJ Open* 2021;11(4):e049210. doi: 10.1136/bmjopen-2021-049210 [published Online First: 20210422]

13. Seuren LM, Wherton J, Greenhalgh T, et al. Physical Examinations via Video for Patients With Heart Failure: Qualitative Study Using Conversation Analysis. *J Med Internet Res* 2020;22(2):e16694. doi: 10.2196/16694

For peer review only - http://bmjopen.bmj.com/site/about/guidelines.xhtml

14. Brown F, Grey E, Sanders I, et al. “A disembodied voice over the telephone”: A qualitative study of healthcare practitioners’ experiences in geriatric medicine. *BMC Geriatrics* In press

15. Braun V, Clarke V. Using thematic analysis in psychology. *Qualitative research in psychology* 2006;3(2):77-101.

16. Braun V, Clarke V, Hayfield N, et al. Thematic analysis. 2019:843-60.

17. Ritchie J, Lewis J, McNaughton Nicholls C, et al. Qualitative Research Practice. 2nd ed. UK: Sage 2014.

18. Gilbert AW, Jones J, Stokes M, et al. Patient, clinician and manager experience of the accelerated implementation of virtual consultations following COVID-19: A qualitative study of preferences in a tertiary orthopaedic rehabilitation setting. *Health Expectations* 2022;25(2):775-90. doi: https://doi.org/10.1111/hex.13425

19. Dhahri AA, Iqbal MR, Pardoe H. Agile Application of Video Telemedicine During the COVID-19 Pandemic. *Cureus* 2020;12(11):e11320. doi: 10.7759/cureus.11320 [published Online First: 20201104]

20. Flodgren G, Rachas A, Farmer AJ, et al. Interactive telemedicine: effects on professional practice and health care outcomes. *Cochrane Database Syst Rev* 2015;2015(9):Cd002098. doi: 10.1002/14651858.CD002098.pub2 [published Online First: 20150907]

21. Mann C, Turner A, Salisbury C. The impact of remote consultations on personalised care: Evidence briefing. Available from: https://arc-w.nihr.ac.uk/Wordpress/wp-content/uploads/2021/08/Remote-consultation-briefing-website-final.pdf: NIHR ARC West, 2021.

22. Chan A, Suarez A, Kitchen J, et al. Teleclinics in rheumatology introduced during the first lockdown phase of the COVID-19 pandemic of 2020. *Future Healthcare Journal* 2021;8(1):e27-e31. doi: 10.7861/fhj.2020-0093

For peer review only

23. Harrison R, Walton M, Chitkara U, et al. Beyond translation: Engaging with culturally and linguistically diverse consumers. *Health Expectations* 2020;23(1):159-68. doi: https://doi.org/10.1111/hex.12984

24. Clarke MA, Fruhling AL, Sitorius M, et al. Impact of Age on Patients' Communication and Technology Preferences in the Era of Meaningful Use: Mixed Methods Study. *J Med Internet Res* 2020;22(6):e13470. doi: 10.2196/13470 [published Online First: 20200601]

For peer review only

For peer review only - http://bmjopen.bmj.com/site/about/guidelines.xhtml

Remote consultations- Rheumatology

Interview schedule - patient

Thank you for agreeing to take part in this project. As you'll have read in the information document, we are interested in your experience and views of remote rheumatology consultations. So, in this call, I'll ask you some questions about these consultations – there are no right or wrong answers, we're just interested in your opinions. I'll record what is said in this call so that it can be typed up later, but any information that could identify you, such as names of people or places, will be removed. If at any time during the call you would like to stop, just let me know, and you do not have to answer any questions that you do not feel comfortable with.

Before we begin, do you have any questions?

[Start recorder]

To start with, can you tell me how many remote (telephone) rheumatology consultations you have had (roughly)?

And when did the remote consultations start? How long had you been having rheumatology appointments before the COVID pandemic?

Can you describe for me what typically happened when you attended the clinic for face-to-face consultations?

How does the session begin? Do you do any preparation before the session?

What information do you try to give the clinician?

How does the session end?

Do you feel the length of the sessions was appropriate (initial session = 30 mins, FU = 15 mins)

Can you describe for me what typically happens in your remote consultations?

How does the session begin? Do you do any preparation before the session?

What information do you try to give the clinician?

How does the session end?

What are the most important differences for you?

Did you receive a clinic letter informing you of your remote consultation?

Do you tend to read everything in your clinic letters or focus on, say, the appointment time?

What, if anything, have you found difficult in conducting remote consultations?

Expressing what is wrong?

Understanding instructions/advice?

Building rapport with clinician?

For peer review only

Technology difficulties – poor lines, hard to take videos of problem area etc.

Has anything helped you to overcome these difficulties?

For example, has asking certain questions helped you to gain a better understanding of what the clinician needs to know/your condition?

Support from partner/friend during the call?

Has anything about the remote consultation been better for you than when meeting in person?

Convenience? More relaxed at home? Not having to take time off work? No travel/parking costs? No waiting at the hospital?

Have you had any face-to-face consultations recently/since having remote consultations?

If yes, how did you find these?

Would you have any concerns if the clinic were to keep running consultations remotely (beyond COVID), at least some of the time?

For your level of care or safety?

Are some types of consultations better conducted remotely than others?

If the clinic was to start using video call, rather than telephone, for remote consultations, how would you feel about this?

If there are times when your remote consultation works particularly well, what do you think made the difference?

And what sorts of things make you feel a consultation hasn't gone very well?

Are there any situations where you would prefer a remote consultation?

For example, drug education, disease education, medication review.

Is there any support or training, for clinicians, other staff or for you, that you think would improve remote consultations?

For example, access to more advanced technology, guidance for clinicians on effective consultations?

That's all my questions, is there anything else you would like to add?

Thank them for their time and stop recorder.

For peer review only

Interview schedule - clinician

Thank you for agreeing to take part in this project. As you'll have read in the information document, we are interested in your experience and views of remote rheumatology consultations. So, in this call, I'll ask you some questions about these consultations – there are no right or wrong answers, we're just interested in your opinions. I'll record what is said in this call so that it can be typed up later, but any information that could identify you, such as names of people or places, will be removed. If at any time during the call you would like to stop, just let me know, and you do not have to answer any questions that you do not feel comfortable with.

Before we begin, do you have any questions?

[Start recorder]

To start with, can you tell me how you used to conduct typical face-to-face outpatient consultations?

What preparation would you do before the consultation? (e.g. look at PROMs, screening responses)

How would you start the consultation?

What information would you try to gather from the patient? (likely to differ depending on reason for consultation – what are the differences?)

Are there any visual clues you look for in a face-to-face consultation, e.g., in the way a patient walks into the room or sits down?

How would you try to end the consultation?

Can you tell me how you would conduct a typical remote consultation?

What preparation would you do before the consultation?

How would you start the consultation?

What information would you try to gather from the patient?

How would you try to end the consultation?

What do you feel are the main differences between remote and typical face-to-face consultations from your perspective?

Are there differences in length between remote and typical face-to-face consultations?

Have you noticed any differences in how patients respond in remote versus face-to-face consultations?

What, if anything, have you found difficult in conducting remote consultations?

Inability to conduct clinical examination or conduct further tests?

Rapport building? Expressing empathy? Breaking bad news?

For peer review only

Understanding patients' descriptions/building a clear picture of the problem without being able to see properly? Assessing whether patients have understood the conversation?

Certain types of conditions or patient groups unsuitable for remote consultations?

Technology difficulties – poor lines, fuzzy pictures etc

Has anything helped you to overcome these difficulties or to improve your remote consultations?

Asking certain questions helped you to gain a better understanding of the patients' conditions?

Have you asked patients to perform their own clinical examinations, e.g. using the DAS (disease activity score) app?

Technology support improved connections?

Discussing or debriefing with colleagues about cases or approaches using this format?

How have you found the use of PROMs to assess patients' suitability for telephone consultations?

Do they provide sufficient information?

Any concerns?

Are there any advantages to remote consultations compared with consultations run in person?

Time saving? Patients more relaxed/comfortable in their own homes?

Would you have any concerns about the rheumatology department continuing with, and potentially increasing the amount of, remote consultations in the long-term?

For patient care, confidentiality or safety?

For clinical workload? Emotional exhaustion?

Would you be happy for more consultations to be conducted remotely in future (beyond the COVID pandemic)? Why/why not? Any provisos?

What do you think are the markers of a successful or good quality remote consultation?

Do these differ from the markers of a successful face-to-face consultation? How?

And what would characterise a poor quality remote consultation?

What outcomes do you think it is important to measure during/after remote consultations? Why?

e.g. number of painful joints/the sound of the person's voice in terms of engagement/energy levels/concern about symptom control or questions raised by the patient about ongoing care

Is there any support or training, for clinicians or patients, that you think would improve the quality of remote consultations?

Are the protocols you have for face-to-face appointments (e.g., breaking bad news etc) fit for purpose with remote consultations?

For peer review only

Help for patients in setting up video-call equipment, access to more advanced technology, guidance for clinicians on effective consultations?

That's all my questions, is there anything else you would like to add?

Thank them for their time and stop recorder.

For peer review only

Standards for Reporting Qualitative Research - checklist

SRQR item	Manuscript page number (line number, if applicable)
Item 1. Title	1 (1-2)
Item 2. Abstract	2-3 (1-23, 1-2)
Item 3. Problem Formulation	5
Item 4. Purpose or research question	5 (21-23)
Item 5. Qualitative approach and research paradigm	6-8
Item 6. Researcher characteristics and reflexivity	8-9
Item 7. Context: Setting/site and salient contextual factors; rationale	5 (15-23) and 6 -7 (18-2)
Item 8. Sampling strategy	7
Item 9. Ethical issues pertaining to human subjects	6
Item 10. Data collection methods	7-8
Item 11. Data collection instruments and technologies	7-8, plus Supplementary file 1
Item 12. Units of study	8
Item 13. Data processing	8
Item 14. Data analysis	8
Item 15. Techniques to enhance trustworthiness	8
Item 16. Synthesis and interpretation	9 - 18
Item 17. Links to empirical data	Throughout Discussion, pages 18-23
Item 18. Integration with prior work, implications, transferability, and contribution(s) to the field	Throughout Discussion, pages 18-23
Item 19. Limitations	22
Item 20. Conflicts of interest	None to declare, 23 (20)
Item 21. Funding	23 (22)

For peer review only
